# Complex Seizure Disorder Caused by *Brunol4* Deficiency in Mice

**DOI:** 10.1371/journal.pgen.0030124

**Published:** 2007-07-27

**Authors:** Yan Yang, Connie L Mahaffey, Nathalie Bérubé, Terry P Maddatu, Gregory A Cox, Wayne N Frankel

**Affiliations:** The Jackson Laboratory, Bar Harbor, Maine, United States of America; Stanford University School of Medicine, United States of America

## Abstract

Idiopathic epilepsy is a common human disorder with a strong genetic component, usually exhibiting complex inheritance. We describe a new mouse mutation in C57BL/6J mice, called frequent-flyer *(Ff),* in which disruption of the gene encoding RNA-binding protein Bruno-like 4 *(Brunol4)* leads to limbic and severe tonic–clonic seizures in heterozygous mutants beginning in their third month. Younger heterozygous adults have a reduced seizure threshold. Although homozygotes do not survive well on the C57BL/6J background, on mixed backgrounds homozygotes and some heterozygotes also display spike-wave discharges, the electroencephalographic manifestation of absence epilepsy. *Brunol4* is widely expressed in the brain with enrichment in the hippocampus. Gene expression profiling and subsequent analysis revealed the down-regulation of at least four RNA molecules encoding proteins known to be involved in neuroexcitability, particularly in mutant hippocampus. Genetic and phenotypic assessment suggests that *Brunol4* deficiency in mice results in a complex seizure phenotype, likely due to the coordinate dysregulation of several molecules, providing a unique new animal model of epilepsy that mimics the complex genetic architecture of common disease.

## Author Summary

Epilepsy is a very common brain disorder characterized by recurrent seizures, resulting from abnormal nerve cell activity in the brain. Some cases of epilepsy are caused by brain trauma, such as stroke, infection, tumor, or head injury. Others—so called “idiopathic”—do not have a clear cause. Many idiopathic epilepsies run in families, but the inheritance patterns and complex seizure types suggest that they are not due to a single defective gene but instead are caused by multiple gene defects that are inherited simultaneously in a patient. This complex inheritance makes it difficult to pinpoint the underlying defects. Here, we describe a new mutant mouse, called “frequent-flyer,” which has several different types of seizures. Although these seizures are caused by a mutation in a single gene, because this gene regulates the expression of many other genes, which, in turn, cause abnormal nerve cell activity, frequent-flyer mice provide a unique animal model of epilepsy—mimicking the complex genetic architecture of common disease.

## Introduction

Epilepsy, defined by recurrent seizures resulting from abnormal, synchronized neuronal firing in the brain, is a very common neurological disorder. Idiopathic epilepsies do not have any antecedent disease or injury to the brain and many are suspected to have a genetic basis. The difficulty of elucidating defective genes underlying common inherited epilepsies is that they are genetically complex—being caused by multiple variants that are coinherited in affected individuals [1,2]. To date, most mutations involved in idiopathic epilepsy have been found in genes encoding ion channels or their accessory subunits with a few exceptions, for example, *LGI1* [3] and *EFHC1* [4] in humans, [5] and *JRK/JH8* [6,7] in both humans and mice. Such exceptions are of interest in that they may lead to further understanding of epilepsy disease mechanisms beyond primary excitability defects, for example, by identification of genes that modulate the expression or function of the more proximal candidates for epilepsy—ion channels, neurotransmitter receptors, and synaptic proteins.

Here we describe the disruption of the expression of an RNA-binding protein, BRUNOL4 (Bruno-like 4) leading to partial limbic and tonic–clonic seizures in a new mouse model of epilepsy called “frequent-flyer” (abbreviated *Ff;* gene symbol: *Brunol4* ^Ff^). BRUNOL4 (also known as CELF4, CUG-BP, and ETR-3 like factor 4) belongs to a family of RNA-binding proteins involved in multiple aspects of RNA processing such as pre-mRNA splicing [8], mRNA editing [9], and RNA stability and translation [10]. There are six family members in both humans and mice with orthologs in nematode and fruit fly [11]. The murine and human BRUNOL4 are 99.6% identical at the amino acid level [12]. Mouse knockouts have very recently been published for Brunol1 and Brunol2, which display spermatagonial and varied developmental defects, respectively [13,14]. UNC-75, a neuron-specific ortholog in C. elegans, shares 47% identity with the human BRUNOL4 protein. UNC-75 deficiency in the nematode leads to behavioral phenotypes indicative of abnormal neurotransmission. Human *BRUNOL4* can rescue the *unc-75* mutant phenotype, suggesting that UNC-75 and BRUNOL4 may be involved in fine-tuning synaptic transmission through regulating RNA processing in the nervous system [15].

In this study we describe the seizure phenotypes of mice carrying *Brunol4* disruption, and begin to explore the molecular consequences using gene expression profiling and genetic interaction tests. Our studies suggest that *Brunol4* deficiency alters the expression of several molecules involved in synaptic function, which, when combined, account for the complex seizure disorder of frequent-flyer mice.

## Results

### Origin of the Mutation and Convulsive Seizure Phenotype

The *Ff* mutation arose from an independent project in which a series of transgenic mouse lines was generated on the C57BL/6J (B6) strain background. One line (9/9 transgene carriers) developed frequent seizures from about three months of age, precipitated by routine handling such as cage transfer. Since the transgene construct was not expressed in all the lines and since other lines using the same construct did not have seizures, together suggested that the seizures were not caused by transgene expression *per se*. To distinguish between an unlinked spontaneous mutation and insertional mutagenesis, affected mice were outcrossed to normal B6 mice. In the next generation, seizures cosegregated with the presence of the transgene (25/28 carriers displayed seizures versus 0/22 non-carriers), suggesting a high-penetrance, dominant mode of inheritance for the seizure phenotype.

**Figure 1:**
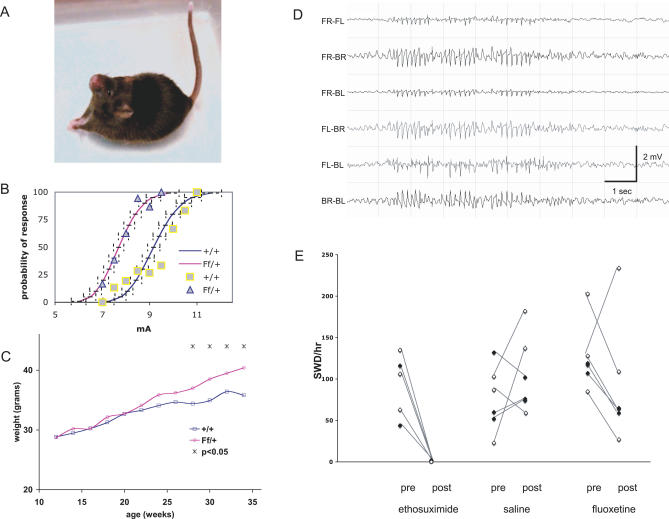
Phenotypes of *Ff* Mutant Mice (A) The posture of a 3-mo-old *Ff/+* heterozygote mouse at the beginning of a seizure episode. Note the contraction of the muscles of the neck, ears, and forelimbs and the vertical position of the tail. (B) Reduced ECT of *Ff/+* mice. The median response level (CC_50_) for minimal seizures were as follows +/+ = 9.17 mA (95% CI 8.83–9.44), *Ff*/+ = 7.69 mA (95% CI 7.48–7.9). Squares (□) and triangles (▵) indicate individual data points used to construct CC curves in +/+ and *Ff/+* mice, respectively. (C) Late-onset body-weight gain in *Ff/+* heterozygous mice. We monitored the body weight between the age of 12 wk and 34 wk of more than 50 male mice with similar number of mutants and controls. Single-caged mice were excluded from this study. Each mouse was weighed every other week. The body weights of mutants did not diverge significantly from those of controls until 28 wk of age and the divergence remained afterwards (*t*-test, assuming two-tailed distribution and two-sample equal variance, the *p-*values are 0.024, 0.004, 0.0011, and 0.001 from 28 wk to 34 wk). The average weight of each genotype at a fixed time-point was plotted against time to generate a growth curve using Microsoft Excel (http://www.microsoft.com/). (D) EEG recording from a *Ff/Ff* homozygous mouse. Shown are the six differential recordings from four subcranial electrodes, one in each quadrant corresponding to front-right (FR), front-left (FL), back-right (BR), and back-left (BL). These traces correspond to one of the longer spike-wave discharge (SWD) episodes observed. (E) Treatment of SWD in *Ff/Ff* homozygous F2 hybrid mice from crosses to FVB (white box) or 129S1 (black). Recording sessions began at least 1 h prior to ethosuximide, saline, or fluoxetine (Prozac) treatment, and the rate of SWD (minimum criteria: ≥ 0.5 s duration, amplitude ≥2× baseline) was determined and plotted (“pre”). Animals were then injected with drug, monitored for an additional hour, and SWD incidence was recorded (post). Lines between datapoints correspond to each animal pre- and post-treatment. Only the ethosuximide and fluoxetine results were significantly different comparing treatments (ETX, *p* = 0.009; fluoxetine, *p* = 0.019; saline, *p* = 0.804 matched pairs test).

Convulsive seizures ranged in severity, the mildest being muscle twitching in the face and neck, forelimb clonus, and salivation (Figure 1A). More severe seizures included rearing and falling, myoclonic jerks, and arching of the back and tail. In many cases, convulsions were followed by a very wild running–bouncing phase with occasional tonic–clonic hindlimb extension, but which, unlike the equivalent phase of some induced seizures, did not result in lethality. Hence, the allele symbol “frequent-flyer” *(Ff)* was assigned. The incidence of these handling-associated seizures was higher in male than in female mice.

Although handling-associated seizures did not begin until the third month of age, by 7 wk heterozygotes had markedly reduced electroconvulsive thresholds (ECT) (Figure 1B). In addition to convulsive seizure phenotypes, heterozygotes were also slightly hyperactive, and while slightly smaller at weaning age, they had a late-onset body weight gain in *Ff/+* heterozygotes (on average 10% heavier than littermate controls, Figure 1C). Despite the high frequency and the severity of seizures, *Ff/+* heterozygotes do not have a reduced life span (analyzed up to 24 mo of age). The morphology of the *Ff/+* brain appears normal, as evident in the proper cortical and hippocampal layering and the lack of overt gliosis (unpublished data).

### Strain Background Effects on Frequent-Flyer Phenotypes

*Ff/Ff* homozygotes, however, had a much more severe phenotype; they were born alive at close to Mendelian ratios but most died during the first day. From matings between heterozygotes, only 1.1% (expect 25%) survived until 4 wk of age (Table 1). While alive, homozygotes did not display obvious signs of convulsion or respiratory stress, nor was there any obvious pathology seen in mutant brains (unpublished data). Future work will be needed to clarify the cause of perinatal lethality in *Ff/Ff* homozygotes. However, when we examined the F_2_ generation of matings between B6-*Ff*/+ and six different inbred mouse strains, a range of survival rates of homozygotes were observed was with the highest being 8.2% in crosses with 129S1 (Table 1), suggesting that homozygosity for B6 allele(s) makes the homozygous phenotype worse, as is the case with many neurological mutations in mice (e.g., see [16]). Interestingly, although these F_2_ hybrid homozygotes often lived for more than 6 mo, they were smaller than littermates and also exhibited spontaneous limbic and tonic–clonic seizures, similar in appearance to those of *Ff/+* heterozygotes, except they were observed as early as 8 wk (and we suspect that lethal seizures occurred as early as 4 wk). In addition, *Ff*/+ heterozygotes on F_1_ hybrid backgrounds experienced a lower incidence of convulsive seizures later in life (unpublished data). These results show that inbred strains have polymorphisms that attenuate the frequent-flyer phenotypes.

**Table 1:**
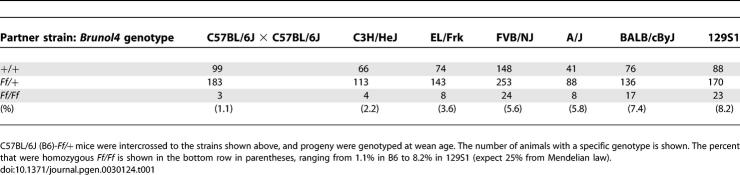
Survival of *Brunol4^Ff/Ff^* Homozygous Mutants in F_2_ Generation

### Absence Epilepsy in Frequent-Flyer Mutants

The availability of *Ff/Ff* homozygotes on a mixed genetic background afforded us the opportunity to determine whether they show spike-wave discharges (SWD), the electroencephalographic manifestation of absence seizures—events not observed in *Ff/+* heterozygotes on the B6 background (unpublished data). *Ff/Ff* homozygotes tested on the F_2_ hybrid backgrounds (B6 × 129S1 or FVB/NJ) experienced very frequent SWD (e.g., see Figure 1D and 1E). Interestingly, heterozygotes in the FVB/NJ cross, but not in the 129S1 cross, also showed a significant rate of SWD (unpublished data). Together, these results suggest that not only do *Ff/Ff* homozygotes have SWD, but that the penetrance or severity is also modulated by genetic background. Although SWD in *Ff/Ff* homozygotes were synchronous, rhythmic, and generalized, when compared to those of other SWD-prone mice, such as stargazer or C3H/HeJ (e.g., see [17,18]), the episodes were relatively short, averaging 1.5 s in length, and the rhythmicity was more erratic than in other mutants (Figure 1D). Nevertheless, the animals remained motionless during SWD episodes, and SWD were suppressed by the anti-absence drug ethosuximide (Figure 1E, left), suggesting that they are absence seizures.

### Transgenic Insertion Mutation in *Brunol4*

**Figure 2:**
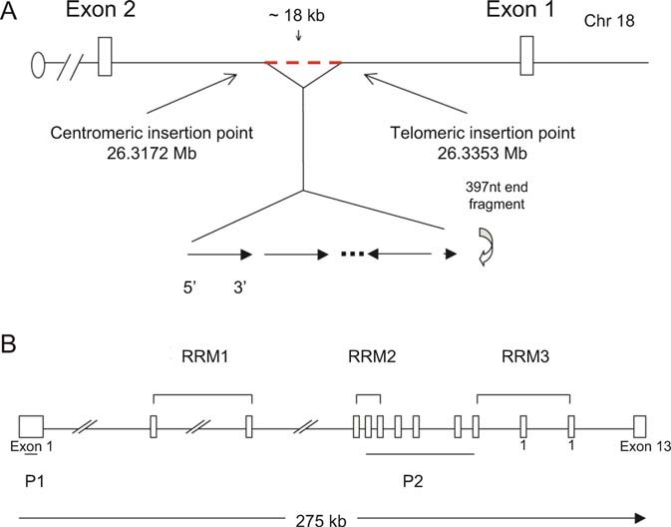
Trangene Insertion into the *Brunol4* Locus in *Ff* Mice (A) Physical map of the transgene insertion point in the first intron of *Brunol4* gene on mouse Chromosome 18. The exact number of copies of the transgene inserted is currently unknown. It appears to be more than four copies based on a Southern blot probed with a transgene-specific probe. The transgene insertion event was a simple addition at the 5′ end, but appeared complex at the 3′ end. The last intact copy of the transgene was inverted to the opposite orientation and there was an 18-kb deletion in the genomic sequence at the 3′ insertion site. Further, a 397-nt fragment from the transgene was left between the last intact copy of the transgene and the intronic sequence from *Brunol4*. This 397-nt fragment was in the same orientation as that of the transgene at the 5′ insertion site. The telomeric breakpoint of the transgene insertion was 28,987 nt from the exon 1 splice donor and the centromeric breakpoint was 27,056 nt from the exon 2 splice acceptor. (B) The exon/intron structure of the mouse *Brunol4* gene. Note the orientation of the gene is reversed from (A) to facilitate viewing. The open reading frame starts in exon 1 and ends in exon 12. Three RNA recognition motifs are predicted in the BRUNOL4 peptide sequence and the respective coding exons are marked.

**Figure 3:**
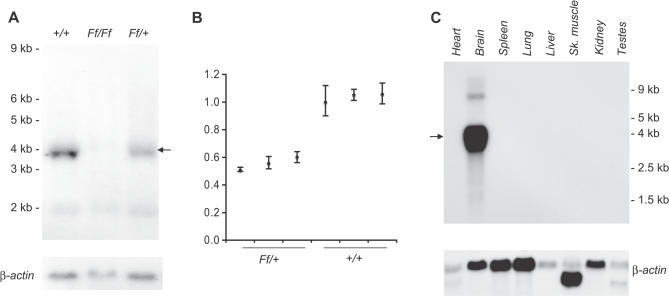
Expression of *Brunol4* in Mutant and Wild-Type Mice (A) Reduced *Brunol4* transcript abundance in *Ff/+* mice. Newborn brain total RNA blot was probed with *Brunol4* p1 probe (upper portion) and a mouse β-actin probe (lower portion) sequentially. *Brunol4* transcript was absent from *Ff/Ff* homozygous newborns, and reduced in abundance in littermate +/+ controls. An arrow indicates the position of the *Brunol4* transcript. (B) Relative fold change of *Brunol4* by real-time RT-PCR) shown in total RNA from adult cortex in three independent mice of each genotype. Similar results were observed from all other brain regions. (C) The expression of *Brunol4* is restricted to the brain in adult mice. An RNA blot containing poly-A selected RNA extracted from various adult mouse tissues was sequentially hybridized with probes specific to *Brunol4* and β-actin as a loading control. The position of the size markers (Ambion, http://www.ambion.com) is shown to the left side of panels.

To determine the identity of the gene disrupted by transgenic insertion, we cloned and sequenced a unique transgene-genomic junction fragment (see Materials and Methods) and found a 100% match to intron 1 of the *Brunol4* gene on mouse Chr 18. We then evaluated the impact of transgene insertion on *Brunol4* expression. The insertion expanded the 74-kb intron 1 of *Brunol4* by at least 20 kb, well upstream of the exons encoding RNA binding motifs (www.ensembl.org/Mus_musculus, Figure 2). Multiple splice donor and acceptor sites were detected in the transgene, suggesting the possibility of the insertion interfering with normal *Brunol4* splicing. In total RNA samples from newborn mice, no *Brunol4* transcript was detected in *Ff/Ff* homozygotes and approximately 45% reduction was seen in heterozygotes (Figure 3A); by real-time reverse-transcriptase PCR (RT-PCR), similar reduction was observed in the adult brain of *Ff*/+ heterozygotes (Figure 3B). We also examined the potential impact of the transgene on the expression of the neighboring genes. The genomic region where murine *Brunol4* resides is gene poor. Genes with strong annotation are at least 0.5 Mb either 5′ or 3′ away from *Brunol4*. Expression analysis of the neighboring genes with potential brain function did not reveal difference between *Ff/Ff* homozygous mutants and normal controls (unpublished data), suggesting that *Brunol4* is the only brain-expressed gene affected by the transgene insertion. This is consistent with preliminary assessment of a gene-targeted null allele of *Brunol4* that we made recently, which displays handling-associated seizures in older adults, resembling those of frequent-flyer mice with both limbic and wild tonic-clonic phases; younger heterozygotes also have an unusually low threshold to electroconvulsion (C. L. Mahaffey, W. N. Frankel, unpublished results).

**Figure 4:**
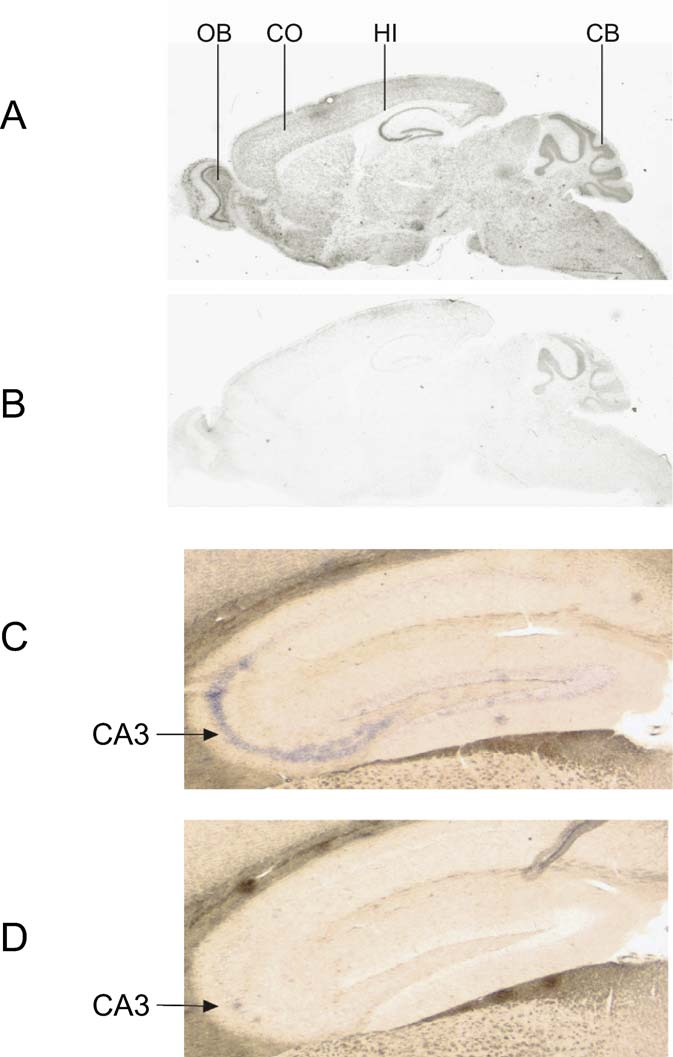
*Brunol4* Expression in Normal Adult Mouse Brain by RNA In Situ Hybridization A ^33^P labeled antisense riboprobe (A) or a sense control probe (B) was hybridized to 7-μm parasagittal paraffin sections of brain. Note the positive staining in the olfactory bulb, cerebral cortex, hippocampus, and cerebellum. Despite several attempts, persistent minor background signal was observed at the edges of the brain section, especially in the cerebellum. In order to increase the specificity and better monitor the signal development, a DIG-labeled antisense riboprobe (C) or a sense control probe (D) was hybridized to 15-μm parasagittal cryosections of brain. Representative images are shown focusing on the entire hippocampus. Note the intense labeling (blue color) in the CA2 and CA3 regions, consistent with the strong hipocampal signal seen in panel (A). CB, cerebellum; CO, cerebral cortex; Hi, hippocampus; OB, olfactory bulb.

In order to evaluate the expression pattern of *Brunol4* in adults, we examined RNA from a variety of tissues. Only brain samples showed robust signal, despite prolonged exposure time, suggesting that BRUNOL4 is brain specific in adults (Figure 3C), consistent with a recent survey of organ protein expression in mice that detected BRUNOL4 only in the brain [19]. At a higher resolution, *Brunol4* showed a predominantly neuronal expression pattern in the brain—labeling was seen in the cerebral cortex, hippocampus, olfactory bulbs, and the granule cell layer of the cerebellum (Figure 4). However, strongest expression in the brain was observed in the hippocampus where high expression was detected in the pyramidal neurons of the CA2 and CA3 region. Pyramidal neurons in CA1, the dentate gyrus granule cells, and the dentate subgranular zone had weaker expression (Figure 4); the latter is interesting in light of the observation that some types of seizure activity may induce neurogenesis in this region (e.g., see [20]).

### Neuroexcitability Candidates Down-Regulated in Mutant Mice

How might BRUNOL4 deficiency result in a complex seizure phenotype? We hypothesize that BRUNOL4 is involved in the processing events of one or more mRNA-encoding proteins that are themselves more directly involved in synaptic function. Thus, in the absence or reduction of BRUNOL4, these molecules become dysregulated, leading to imbalance in neuronal excitability. We carried out microarray analysis to detect genes differentially expressed between coisogenic wild-type and mutant mice. For the primary screen, newborn *Ff/Ff* homozygous mutants were chosen to optimize the signal differential. Of the approximately 39,000 transcripts interrogated, changes in only 459 transcripts (corresponding to approximately 350 independent genes) were considered statistically significant in the *Ff/Ff* homozygotes compared with controls (Table S1). Of the 94 down-regulated transcripts (from approximately 70 independent genes), the most reduced was *Brunol4* itself, with a significant decrease in *Ff/Ff* homozygotes. Four genes from the down-regulated list were of obvious interest for an excitability disorder. One encodes serotonin receptor 2c *(Htr2c); Htr2c*-null mice are known to experience frequent spontaneous seizures, at least on a mixed background, as well as a reduced seizure threshold [21]. The second encodes synapsin II *(Syn2);* seizures precipitated by sensory stimuli were found previously in *Syn2*-knockout mice [22]. The third encodes N-ethylmaleimide-sensitive factor *(Nsf),* which regulates exocytosis in synaptic transmission, as well as AMPA receptor trafficking [23,24]. The fourth encodes α-synuclein *(Snca),* a neuron-specific presynaptic protein [25]. All four molecules have been found in the pyramidal neurons in the hippocampus where *Brunol4* is highly expressed [25–28]. In particular, the expression of NSF [27] and synapsin II [28] were enriched in the CA2-CA3 region, similar to that of *Brunol4*.

**Figure 5:**
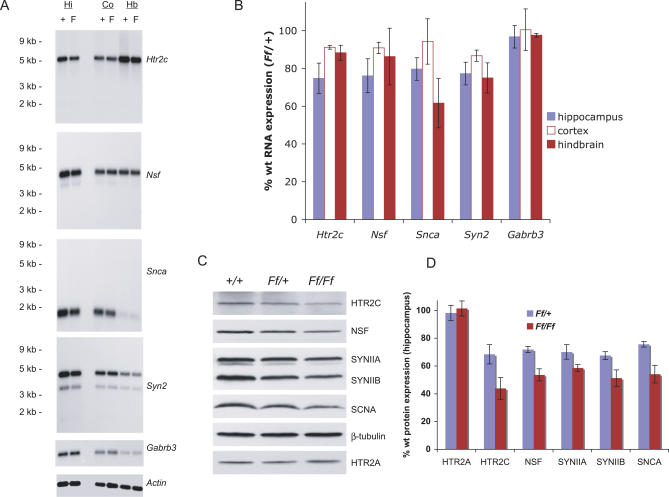
Reduced Expression of Four Candidate Genes in *Ff* /+ Brains (A) Representative images from three independent RNA blots. Sequential hybridization using *Htr2c*, *Nsf*, *Snca*, *Syn2*, *Gabrb3,* and β-actin probes was carried out on a blot containing poly-A RNA extracted from dissected brain regions. The synapsin II probe detected both synapsin IIa and IIb transcripts due to alternative splicing/polyadenylation [40] and there was no overt change in the synapsin 2a/2b ratio between *Ff/+* and *+/+* brains. In addition to the loading control actin, we examined the expression of *Gabrb3,* which encodes the β3 subunit of the GABA(A) receptor. Germline targeting of *Gabrb3* caused perinatal lethality in homozygous mice and overt seizures in heterozygotes [41]. No significant expression difference was found between *Ff/+* and *+/+* brains. The position of size markers (Ambion) was shown on the left. CO, cerebral cortex; Hb, hindbrain; Hi, hippocampus. F, *Ff/+*; +, *+/+*. (B) Histogram summarizing results from northern blots, expressed as percent of wild type after normalization to β-actin. (C) Reduced expression at the protein level; Western blot for HTR2A, HTR2C, NSF, synapsin II, α-synuclein, and β-tubulin. HTR2A, a molecule with distribution and property similar to HTR2C, was used as a control in addition to β-tubulin. Representative images from experiments with three independent adult mice on a mixed background are shown. (D) Histogram summarizing results from (C) in *Ff/+* and *Ff/Ff* mutants on a mixed background, expressed as % of wild type after normalization to β-tubulin, summarizing data from three independent animals. Error bars throughout are one standard deviation.

After confirming the differential expression of all four transcripts in *Ff/Ff*-homozygous newborn mice (unpublished data), expression levels were assessed in adult *Ff/+* heterozygous brain regions. All four RNAs had, on average, a 20%–25% reduction in the B6-*Ff*/+ hippocampus compared with controls (Figure 5A and 5B). Moreover, these candidates showed a significant reduction at the protein level in adult hippocampus of B6-*Ff/+* mice (30%–39%; unpublished data), and on a mixed background in *Ff/+* and *Ff/Ff*, (25%–32% *Ff/+;* 32%–56% *Ff/Ff,* Figure 5C and 5D). These region-specific decreases before the onset of seizures were consistent with the limbic-seizure phenotype and the overlapping hippocampal expression of the four genes with that of *Brunol4*.

### *Htr2c* Deficiency Contributes to, but Is Not Sufficient for, Seizure Phenotypes in *Brunol4^Ff^* Mutants

**Figure 6:**
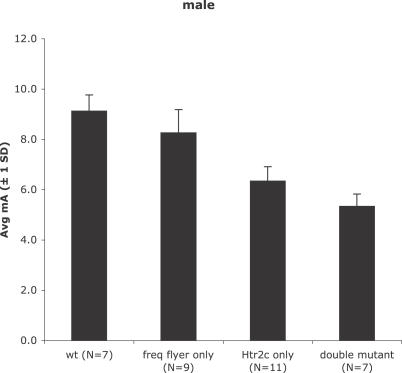
Genetic Interaction between *Brunol4* and *Htr2c* for ECT Shown is a histogram of average threshold (in mA) to a minimal clonic or maximal tonic hindlimb extension seizure, determined as described in Materials and Methods, for wild-type (+/+, +/+), single mutant (*Ff*/+, +/+ or B6-*Htr2c^tm1Jul^*/Y), or double mutant (*Ff*/+; *Htr2c^tm1Jul^*/Y), littermates from crosses between B6-*Htr2c^tm1Jul^*/Y and B6-*Ff*/+ mice.

Null mutants of the *Htr2c* receptor gene have several phenotypic similarities with *Brunol4^Ff^* ^/+^ heterozygotes, including reduced seizure threshold, hyperactivity, and late-onset weight gain [21,29]. However, the fact that *Brunol4^Ff/+^* mice on the B6 strain background experience frequent handling-provoked convulsive seizures after 3 mo of age, whereas *Htr2c* null mutants do not (Y. Yang, W. Frankel, unpublished data), suggests that down-regulating *Htr2c* receptor expression is not sufficient to account for the handling-induced seizures. To determine whether *Htr2c* deficiency is sufficient to account for the ECT of *Brunol4^Ff/+^* mice, we compared seizure threshold in wild-type, single-mutant, and double-mutant mice (Figure 6). These studies were all done on the B6 strain, to avoid the confounding effect of genetic background. Although the average seizure threshold of *Htr2c* null mutants was lower than that of *Brunol4^Ff/+^* mice by approximately 2 mA, the threshold of double mutants was 1 mA lower still (*p* = 0.0003; |*t*|-test). This suggests that factors in addition to reduced expression of *Htr2c* contribute to seizure threshold in *Brunol4^Ff/+^* mice. Another difference between *Htr2c* and *Brunol4* mutant mice is that *Htr2c*-null mutants do not have SWD ([21]; our unpublished observations). However, we found that blocking serotonin reuptake in *Brunol4^Ff/Ff^* homozygotes by fluoxetine (Prozac) treatment lowers the SWD incidence by about 50% (Figure 1E, right)—again suggesting that *Htr2c* down-regulation combines with other Brunol4-downstream deficiencies to cause the seizure disorder of frequent-flyer mutants. Although further tests are required to determine whether the other causative factors are *Syn2*, *Nsf,* or *Snca* specifically, it is clear that the seizure phenotypes of *Brunol4^Ff^* are determined in a genetically complex manner.

## Discussion

Here, we report on the causal association between *Brunol4,* encoding a brain-specific RNA-binding protein, and the seizure disorder of frequent-flyer mouse mutants. The origin of the disorder is a transgenic insertion in the *Brunol4* gene, resulting in very little *Brunol4* transcript in homozygotes and accordingly reduced amount in heterozygotes—suggesting haploinsufficiency. We do not know why the transcript levels were very low, but one possibility is due to the inverted repeat in the transgene cluster, creating a potential hairpin structure that may prevent read-through transcription (Figure 2). *Brunol4^Ff^* mutant mice have several different kinds of seizures, depending upon genotype, age, and strain background. In heterozygotes on a B6 inbred strain background, these include recurrent limbic and tonic–clonic seizures—observed readily following routine animal handling after 3 mo of age—and a significantly lower ECT at an earlier age. Although homozygotes do not usually survive on a C57BL/6J strain background, on F_2_ hybrid backgrounds homozygotes (and some heterozygotes) that survived also displayed spike-wave discharges, the hallmark of absence epilepsy.

The prospect of a defective RNA-binding protein such as BRUNOL4 causing a complex seizure disorder suggests a way in which a single gene defect can mimic a complex genetic disease, by impairing the function of multiple molecules simultaneously. This could happen either as a direct consequence of its absence, or secondarily, e.g., a cascade of effects. Microarray analysis between homozygous mutants and coisogenic control brain yielded a small number of down-regulated transcripts that were statistically significant, two of which *(Htr2c* and *Syn2)* are already known to cause seizure-related phenotypes when knocked out in mice [21,22], and two other *(Nsf* and *Snca)* have obvious functions that relate to synaptic transmission. The down-regulation of each was confirmed in adults, and was greatest in the hippocampus, where *Brunol4* expression is high. Interestingly, in addition to seizure susceptibility, *Ff/+* heterozygotes display two nonseizure phenotypes like those of *Htr2c* null mutants: mild hyperactivity and late-onset obesity [21,30,31]. This might suggest that compromised serotonergic transmission is the major factor of the frequent-flyer phenotype. However, because *Htr2c* expression is reduced only modestly (∼25% RNA, ∼35% protein) in *Ff/+* heterozygotes, compared with complete loss in *Htr2c*-null mice, it seems more likely that a combination of compromised serotonergic transmission and other defects is responsible for the disorder. Further evidence for this idea was obtained in the ECT paradigm (additive phenotypic effects of double mutants), and by observing that SWD phenotype of *Brunol4* homozygotes was partially mitigated by up-regulation of serotonergic transmission through blocking the reuptake of serotonin. With any of the seizure paradigms, it is plausible that reduced synaptic efficacy, e.g., due to reduced expression of the other three candidates singled out—*Syn2*, *Nsf,* or *Snca*—is the other contributing variable. However, we cannot ignore other genes misregulated in *Brunol4* mutants, some with unknown function (Table S1), since many also expressed selectively within the hippocampus in B6 mice (Allen Brain Atlas [32]).

We do not know why *Brunol4* deficiency results in the decreased expression of these and other transcript RNAs. BRUNOL4 has been shown to regulate alternative splicing in cells and tissues without endogenous BRUNOL4 [33–35], but we did not detect aberrant splice variants in mutant brains, at least in the subset of transcripts that we analyzed (our unpublished results). Since members of the Bruno gene family are involved in RNA editing, stability, and translation, the possibility exists that Brunol4 is involved in other aspects of RNA metabolism, for example, in stabilizing transcripts for translation, a possibility that is supported, in part, by the fact that the degree of reduction at the RNA level and at the protein level was not 100% concordant in the mutant hippocampus (∼20%–25% and ∼31%–39%, respectively). However, since many RNA processing steps are believed to be coupled with transcription [36], BRUNOL4 may indeed serve multiple roles in RNA metabolism.

Most genes known to cause idiopathic epilepsy encode ion channels. *Brunol4* joins a growing list of non-ion-channel epilepsy genes in both humans and mice [3–5,7]. It is noteworthy that the human *BRUNOL4* gene is in a region on human Chromosome 18 showing strong evidence for linkage with adolescent-onset idiopathic generalized epilepsy [37], suggesting that *BRUNOL4* may be a candidate gene for these seizure disorders.

## Materials and Methods

### Mice.

Origin of *Brunol4^Ff^* mice. C57BL/6J (B6) transgenic mice were generated at The Jackson Laboratory (http://www.jax.org/) using a construct where the expression of murine *Ighmbp2* cDNA and enhanced green fluorescent protein (EGFP) was driven by a bidirectional tetracycline-responsive promoter. Briefly, the coding region of the *Ighmbp2* cDNA was cloned in the *pBI-EGFP* vector (Clontech, http://www.clontech.com/). The PvuI linearized transgene (∼8.3 kb) was microinjected into pronuclei of single cell B6 mouse embryos, which were subsequently implanted into pseudopregnant mice. B6.129-*Htr2c^tm1Jul^* mice, derived from mice published in 1995 [21], were obtained from The Jackson Laboratory's Induced Mutant Resource and are now fully congenic on the B6 background after backcrossing for ten generations. All animals were fed standard National Institutes of Health diet containing 6% fat and acidified water ad libitum. All animal procedures followed Association for Assessment and Accreditation of Laboratory Animal Care guidelines and were approved by institutional Animal Care and Use Committee.

### ECT.

As previously described [38], mice were restrained, a drop of anesthetic containing 0.5% tetracaine and 0.9% NaCl was placed onto each eye, and a preset current was applied via silver transcorneal electrodes using a electroconvulsive stimulator (Ugo Basile model 7801; http://www.ugobasile.com). The stimulator was set to produce rectangular wave pulses with the following parameters: 299 Hz, 0.2 s duration, 1.6 ms width. Sixty *Ff*/+ and 57 littermate +/+ male mice (ages 6–9 wk) were tested for ECT over a range of electric current settings for minimal clonic forebrain seizure and each ECT response was recorded. The data were analyzed in the computer program MiniTab (Minitab, http://www.minitab.com/) and a response curve was generated using the log-Probit procedure. To determine ECT in double mutants, male mice (ages 6–9 wk) were tested by increasing the stimulus once daily until at least a minimal clonic seizure was observed, and the average threshold determined for each genotype.

### Electroencephalogram analysis.

Mice were anesthetized with tribromoethanol (400mg/kg i.p.) Small burr holes were drilled (1 mm anterior to the bregma and 2 mm posterior to the bregma) on both sides of the skull 2 mm lateral to the midline. Electroencephalogram (EEG) activity was measured by four Teflon-coated silver wires soldered onto a microconnector. The wires were placed between the dura and the brain and a dental cap was then applied. The mice were given a post-operative analgesic of carprofen (5 mg/kg subcutaneous) and were given a 48-h recovery period before recordings were made. The mice were recorded for a 2-h period on each of the following two days using the Grass EEG Model 12 Neurodata Acquisition System and PolyViewPro software program (Grass-Telefactor, http://www.grasstechnologies.com/). For mice that were treated with ethosuximide (200 mg/kg; Sigma-Aldrich, http://www.sigmaaldrich.com) or fluoxetine (20 mg/kg, Sigma-Aldrich), on the day following their second standard EEG recording, mice were recorded for 90 min and then injected intraperitoneally. They were then recorded for a minimum of one additional hour. The control mice were injected intraperitoneally with saline and recorded in the same manner. Matched pairs tests were done using the program JMP 6.0.3 (SAS, http://www.sas.com/).

### Identification of the transgene insertion site.

Genomic DNA from transgenic mice and control mice was digested with BclI, SpeI, BglII, SphI, and StuI and electrophoresed and blotted onto a Nytron Plus membrane (Schleicher & Schuell, http://www.whatman.com/). The blot was probed with an EGFP probe and a unique transgene-genomic junction fragment was present in StuI-digested DNA at about 3 kb. The StuI-digested fragment of ∼3 kb was cloned into the pBluescript II-SK vector (Stratagene, http://www.stratagene.com). A vector-specific primer and an EGFP primer were used to amplify the junction fragment. Automated sequencing confirmed the presence of the EGFP cDNA as well as other vector sequence. A 652-nt fragment did not match with the pBluescript II-SK vector sequence and was used as a query to BLAST search the mouse genome. A single perfect match to intron 1 of the *Brunol4* gene was found on mouse Chromosome 18. The other breakpoint of the transgene insertion was cloned by a PCR strategy using a transgene-specific primer and a *Brunol4* intron 1 primer. Based on the sequence information around the insertion breakpoints in the *Ff* allele, a 3-primer PCR assay was designed to detect the *Ff* allele and wild-type allele. Primers for this assay are: s3gtf, 5′-CTCTTCATCCCTTCTGGCAAGTAG-3′; s3gtr, 5′-GTATTCAACAATTCCGTGTCGCCC-3′; and s3gtr2, 5′-CCACACAGAGACCAAGAAGATTCC-3′. At 55 °C annealing temperature, 35 cycles, standard PCR conditions, the s3gtf/s3gtr2 primers produce a 672-bp wild-type allele, and the s3gtf/s3gtr primers produce a 464-bp mutant allele.

### Northern blot analysis and quantification.

Total RNA was prepared from newborn brain, adult brain and dissected brain regions using TRIzol reagent (Invitrogen, http://www.invitrogen.com). Two probes were generated for *Brunol4*, p1 containing 5′ UTR and exon1 5′ to the insertion site and p2 containing the region between BRUNOL4′s second and third RNA recognition motif. Both probes detected a single transcript on northern blots and p2 was also used for in situ analysis. Hybridization was carried out in formamide-based solution at 42 °C overnight and the blot was washed and exposed to an X-ray film at −80 °C. The same blot was stripped and reprobed with a mouse β-actin probe. Films were imaged by Fuji Luminescent Image Analyzer LAS-1000 Plus (http://fujifilmlifescienceusa.com) and subsequently quantified by Fuji Image Gauge Ver. 3.4.

### Real-time reverse-transcription PCR.

Total RNA was prepared from the cerebral cortex of adult B6-*Ff*/+ and and B6-+/+ /littermates with Trizol (Invitrogen, http://www.invitrogen.com) and treated with DNase I (Promega, http://www.promega.com) under the manufacturer's suggested conditions. RNA (2 μg) was reverse transcribed with AMV reverse transcriptase (Promega). The cDNA was diluted 20-fold, and 1.5 μl was added to qPCR Mastermix Plus for SYBR Green I (Eurogentec, http://www.eurogentec.be) with pairs of the following primers: *beta-actin*F (5′-CATTGCTGACAGGATGCAGAA-3′) and *beta-actin*R (5′-GCCACCGATCCACACAGAGT-3′), *Be1u* (5′-TCGCAGTAGGTGAGGAAAGCGCAG-3′) and *Be2d* (5′-TCGCAGTAGGTGAGGAAAGCGCAG-3′), corresponding to Brunol4 exon 1 forward and exon 2 reverse, respectively. The PCR reactions were analyzed on an ABI Prism 7000 Sequence Detection System (PerkinElmer, http://www.perkinelmer.com/). The PCR amplifications from three pairs of age-matched mice were run in triplicate. Amplification of the correct size products was confirmed by agarose gel electrophoresis.

### RNA in situ hybridization.

Thin brain sections (7 μm) from 10-wk-old B6 male mice were hybridized with ^33^P probes overnight at 50 °C and washed, RNase A treated, dehydrated, and air dried. Slides were dipped in liquid emulsion (Kodak, http://www.kodak.com/) and images were developed 5 d afterwards. For DIG-based in situ analysis, 15-μm cryosections were hybridized with DIG probes overnight at 65 °C and washed extensively before an overnight incubation of alkaline phosphatase–conjugated anti-DIG antibody (1:2,000, Roche, http://www.roche.com/). Staining signal was developed using BM purple (Roche) at room temperature for 12 h.

### Microarray analysis.

Total RNA was prepared from six male newborn heads (3 *Ff/Ff* and 3 *+/+*). 10 μg of total RNA was used to generate 15 μg of cRNA for hybridization to the Affymetrix 430 v2.0 Gene Chip (Affymetrix, http://www.affymetrix.com/) according to manufacturer's recommendation. Using the R/maanova package [39], an analysis of variance (ANOVA) model was applied to the data, and F1, F2, F3, and Fs test statistics were constructed along with their permutation *p*-values. Changes in 459 transcripts were considered statistically significant among the 39,000 transcripts interrogated.

### Antibodies for Western blotting.

The primary antibodies and the dilutions were: α-HTR2A, 1:500 (BD Pharmingen, http://www.bdbiosciences.com/); α-HTR2C, 1:500 (Immunostar, http://www.immunostar.com/); α-NSF, 1:1,000 (H-300, Santa Cruz Biotechnology, http://www.scbt.com/); α-Synapsin 2, 1:5,000 (Stressgen, http://www.nventacorp.com/); α-alpha-synuclein, 1:250 (BD Transduction, http://www.bdbiosciences.com/); and α-beta-tubulin, 1:1,000 (Sigma). The secondary antibodies and the dilutions were: HRP α-mouse, 1:6,000 (Zymed, http://www.invitrogen.com/) and HRP α-rabbit, 1:2,000 (PerkinElmer).

### Northern blot probes.

The following probes were designed to detect expression differences among the putative BRUNOL4 target RNAs between *Ff/+* and *+/+* brains: (1) *Htr2c*, 437-nt probe in exon 6, the last nucleotide is 43 nt 3′ of the TAA stop codon. (2) *Nsf*, 470-nt probe as described in [27]. (3) *Syn2*, 234-nt probe in exon 1, the first nucleotide is 90 nt 3′ of the ATG start codon. This probe was able to detect the two alternatively polyadenylated *Syn2* transcripts. (4) *Snca*, 416-nt probe in exon 6, the first nucleotide is 37 nt 3′ of the TAA stop codon. (5) *Gabrb3*, 412-nt probe specific to the 3′ end of the coding sequence, the last nucleotide is 14 nt 5′ of the TGA stop codon.

### Western blot.

Hippocampi were dissected from three male *Ff/+* mice (before the onset of handling-provoked seizures) and three +/+ littermates. Protein extracts were made using RIPA buffer with proteinase inhibitors (Roche) and subsequently quantified using the Bradford reagent (Bio-Rad, http://www.bio-rad.com/). Protein (50 μg) from each sample was loaded and probed with the primary antibody and a secondary peroxidase-conjugated antibody and visualized with the ECL plus kit (Amersham, http://www.amersham.com/). The nitrocellulose membrane was incubated with Restore Western blot stripping buffer (Pierce, http://www.piercenet.com/) at 37 °C for 30 min to remove all the antibodies. The membrane was washed and subsequently reprobed with a different set of antibodies. Signals were quantified using the method described above.

## Supporting Information

Table S1 List of Genes with Differential Expression between *Ff/Ff* Homozygous and Wild-Type Newborn Mice Microarray analysis was carried out as described in Materials and Methods. Down-regulated transcripts (94) are listed first, followed by 365 up-regulated transcripts.
(416 KB XLS)

### Accession Numbers

The National Center for Biotechnology Information (NCBI) Nucleotide database (http://www.ncbi.nlm.nih.gov/sites/entrez?db=Nucleotide) accession number for intron 1 of the *Brunol4* gene on mouse Chr 18 is EF639873.

## Abbreviations

*Brunol4,*: Bruno-like 4
ECT: electroconvulsive threshold
EEG: electoencephalogram
*Ff,*: frequent-flyer
RT-PCR: reverse-transcriptase PCR
SWD: spike-wave discharge
